# Determining the Effects of a 6-Week Training Intervention on Reactive Strength: A Single-Case Experimental Design Approach

**DOI:** 10.3390/jfmk10020191

**Published:** 2025-05-26

**Authors:** Benjamin Southey, Dirk Spits, Damien Austin, Mark Connick, Emma Beckman

**Affiliations:** 1 School of Human Movements and Nutrition Science, The University of Queensland, St. Lucia, QLD 4067, Australia; b.southey@uq.net.au; 2 Brisbane Lions Australian Football Club, Springfield, QLD 4300, Australia; 3 Tennis Australia, Tennyson, QLD 4105, Australia; 4 School of Exercise and Nutrition Sciences, Queensland University of Technology, Brisbane, QLD 4006, Australia

**Keywords:** 10/5 repeated jump, drop jump, multiple baseline design, strength training, reactive quality ratio

## Abstract

**Objectives**: Single-case experimental designs (SCEDs) provide a robust way to observe adaptations to training in highly specific populations. Furthermore, they provide unique insights into inter-participant variance in responses to interventions, which traditional randomized control trials cannot obtain. However, there is limited sports science literature that has applied this methodology to assess the effectiveness of training interventions. Thus, the aim of this study was to determine the individual and combined changes to reactive strength following a 6-week strength and plyometric training intervention. **Methods**: A non-concurrent multiple baseline SCED was used, where four participants completed weekly 10/5 repeated jump (RJ), drop jump (DJ), and loaded squat jumps during a 5–7-week baseline phase and a 6-week intervention phase. The intervention consisted of traditional resistance and plyometric training. **Results**: The results found inter-participant variance in changes to reactive strength, with some individuals having significant improvements whilst others declined. The combined results found that during the intervention, the reactive strength index (RSI) of the RJ significantly decreased (baseline: 2.15 vs. intervention: 2.0) whilst no change in DJ occurred. This led to a significant increase in the reactive quality ratio (RQR) (baseline: 1.02 vs. intervention: 1.08). **Conclusions**: These findings highlight the importance of considering individual responses to training reactive strength rather than cohort observations alone, and the SCED is a viable methodology to achieve this. Practitioners should consider exercise selection, maximum strength levels and responsiveness of individuals when prescribing plyometric exercise to improve high and low amplitude reactive strength qualities.

## 1. Introduction

Improving an individual’s ability to express force in a reactive jump environment is an important area of focus for strength and conditioning coaches and sports science practitioners, as this force expression is frequently experienced in locomotive sporting tasks [1,2,3,4]. However, when exploring the literature that analyzes training interventions, randomized control trials (RCTs) are often used as a method to observe outcome measures [5,6,7,8,9,10]. Whilst RCTs are robust at determining change at a cohort level, this experimental design cannot statistically determine the effects of a training intervention at an individual level due to the internal variability of each participant within a cohort [11]. This is a particularly important consideration for strength and conditioning coaches and sports science practitioners when wanting to make physical adaptations to individual outliers in large training squads. Therefore, single-case experimental design (SCEDs) can offer a way to bridge that gap. The term SCED is used to describe an overarching category of experimental research design where the use of a single participant serves as their own control, allowing for direct comparisons between baseline and intervention phases to be made [12]. This is carried out by undertaking consistent repeated measure testing on an individual or small group of individuals during a baseline (A), an intervention (B), and a follow-up (return to baseline) phase. Typically, a minimum testing requirement is 3–5 observations per phase, which allows investigators to track the participants’ trends in response to training over time [12]. Research using SCED in sports science has been previously used with paralympic athletes due to their low and unique sample sizes [13,14]. They have, however, been extensively used in rehabilitation and medicine research, with a randomized controlled single-case design being given a rank of level 1 evidence for treatment decision purposes in individual patients by the Oxford Centre for Evidence-Based Medicine [12,15]. Given the scientific rigor, it stands to reason that sports science researchers could consider the utility of the SCED, given the imperative to have strong, specific, individualized outcomes from strength training interventions. However, to the authors’ knowledge, there are no studies that have used this method for such observations in broader populations.

In practice, strength and conditioning coaches will often prescribe low amplitude, extensive plyometrics before progressing to high amplitude, intensive plyometrics. However, the reactive strength index (RSI) during these tasks has been shown to have little shared agreement, indicating unique reactive strength qualities due to the differences in jump strategies and force expression [16]. This means that individuals may benefit from training these plyometric tasks concurrently instead. The drop jump (DJ) is a commonly used high amplitude exercise due to its relationships with maximum strength and higher ground reaction forces, landing rate of force development, and impulse compared to the 10/5 repeated jump (RJ), which is a commonly used low amplitude test [17]. Both tests have excellent reliability in the RSI [18,19,20,21,22,23]. Additionally, the reactive quality ratio (RQR) is a reliable way to compare relative differences between the DJ and RJ RSI. This can be helpful for practitioners in determining whether individuals should spend more time undergoing low and/or high amplitude plyometric training [17]. However, the inter-participant variance in changes to reactive strength following training is unknown. Therefore, the aim of this study was to examine the individual and combined effects of a 6-week strength and plyometric training intervention on reactive strength output and underlying variables using a SCED method. It was hypothesized that following the intervention block, there would be a greater increase in the DJ RSI due to all participants increasing their maximum strength output. Thus, the RQR would also increase as a reflection of a greater change in the DJ RSI than the RJ RSI.

## 2. Materials and Methods

A non-concurrent multiple baseline (NC-MBD) single-case experimental design was used for this study due to the increased statistical power of having multiple participants. Additionally, the NC-MBD was selected as the design of choice as it eliminates the requirement of a follow-up block [12]. The follow-up block is important in ensuring the intervention is not influenced by coincidences, session experiences, or maturation factors [24]. Hence, trends in outcome measures during the follow-up block should return to baseline. However, in an exercise setting, this phase would be significantly impacted by the long-lasting adaptations following training interventions [11]. Therefore, the NC-MBD mitigates this requirement by staggering the start of the intervention block between participants to eliminate the factors that could impact the causality of the intervention [24]. Therefore, participants completed either a baseline block of 5, 6, or 7 weeks before starting the intervention block. Furthermore, participants had different backgrounds and lived and worked in different environments, further controlling for coincidental and environmental impacts on the combined results of the intervention.

During the baseline block, participants completed a weekly testing session where RJ, DJ, and loaded squat jumps were carried out, following a standardized warm-up. During the intervention block, participants completed two weekly lower-body gym sessions comprising of strength and plyometric training; see program (Table 1). Sessions were completed on consistent days of the week across the intervention block. The first gym session of the week also comprised the testing session, which was completed before training. At least two days separated the two gym sessions to provide ample recovery between workouts. All 4 participants who were included in the final analysis completed all 12 training sessions. Participants were to refrain from any additional lower-body strength work during the 11–13-week observation period; participants could continue to complete any regular recreational cardio activities as long as they remained consistent throughout both baseline and intervention phases.

### 2.1. Participants

A total of six male participants were recruited, consented to participate in the study and completed the observation period. Participants were recreationally active, with some previous resistance training experience. However, data from only 4 of the participants were included for final analysis. Data from two participants were withdrawn due to one participant sustaining a minor injury during the intervention phase and the other having a spike in physical workload, leading to inconsistent working hours and fatigue, which occurred during the observation period. Ethics for this study was approved by the university’s ethics committee, application—#2021/HE002747. Details of each participant included in the final analysis are seen below:

Participant 1: Male; age: 24 years; weight: 73 kg—Worked a standard 9 a.m.–5 p.m. corporate job. Recreational gym goer, going inconsistently 1–2 times a week for the previous three years prior to recruitment. No previous injuries were reported. Participant 1 completed a 5-week baseline phase before completing the intervention.

Participant 2: Male; age: 22 years; weight: 67 kg—A mathematics student at university who had little resistance training experience, only going to the gym a half dozen times in the previous 6 months. No previous injuries were reported. Participant 2 completed a 5-week baseline phase.

Participant 3: Male; age: 28 years; weight: 91 kg—An exercise physiology student at university who had previous resistance training experience, training for basketball in his early twenties. This participant had not limited resistance training exposure in the previous 2–3 years. He also had an ankle injury in the previous 6 months before recruitment from playing recreational sports but was healed before the study. Participant 3 completed a 6-week baseline phase.

Participant 4: Male; age: 31 years; weight: 77 kg—Worked a corporate 9 a.m.–5 p.m. job and was recreationally active, running and going to the gym for the past 5 years. However, in the 6 months leading up to recruitment, going to the gym and running were inconsistent, with periods of high activity (2x/wk gym, 2–3x/wk run) and periods of no activity. There were no reported injuries or niggles. Participant 4 completed a 7-week baseline phase.

### 2.2. Outcome Measures

All kinetic and temporal data were collected using a force platform (Vald Performance Force Deck Dual Platform FD4000, Newstead, Queensland, Australia) at a sampling rate of 1000 Hz; capacity: 2000 kg; resolution: c.15 g/0.15 N.

#### 2.2.1. RQR Assessment

The RQR was calculated by dividing the RSI of the DJ by the RSI of the RJ. The RSI was calculated by dividing the flight time (FT) by the ground contact time (GCT) during respective tests.

10/5 Repeated Jump: Participants were cued to stand with their hands on their hips and complete one countermovement jump followed by 10 consecutive pogo hops, emphasizing both maximal jump height and minimal ground contact time. One set was completed, unless an invalid test occurred, in which another trial was conducted. This would happen if an athlete hopped off the force platforms mid-test or lost rhythm to their hops due to movement around the platforms. Of the 10 hops, the 5 best (indicated by the highest RSI) were averaged and used for analysis.

Drop Jump: All participants completed three drop jumps from a box height of 45 cm, and these scores were then averaged and used for analysis. Similar to the RJ, participants were instructed to have their hands on their hips throughout the test and step off the box, emphasizing maximal jump height with minimal ground contact time when landing on the force platforms. Participants were required to complete an extra jump if there was an invalid result, which would occur if participants jumped or stepped down off the box rather than stepped off the box. These strategies would impact the fall height and thus the eccentric demands of the test.

#### 2.2.2. Force–Velocity Profile

Due to the regularity of testing conducted throughout this study, it was considered to not be an optimal training stimulus for participants to conduct weekly maximal strength testing. Therefore, a submaximal strength assessment, such as the jump Force–Velocity Profile (FVP) developed and validated by Samozino [25], was utilized as an outcome measure in this study due to its ability to determine one’s force expression (e.g., theoretical max force, power, and velocity) characteristics [25,26,27]. The FVP was important in providing insights into what underlying strength changes may impact variation in the RQR. A free-to-download FVP spreadsheet created by Samozino and Morin (https://jbmorin.net/2017/10/01/a-spreadsheet-for-jump-force-velocity-power-profiling/, accessed 30 January 2024) was used to calculate an individual’s FVP. Anthropometrical measures of bodyweight (BW), lower limb length (anterior superior iliac crest to tip of toes), and initial height (lower limb length when knees are bent at 90°) were all measured to calculate push-off distance and relative outputs, which were automatically calculated in the spreadsheet. Participants completed 4 sets of 2 reps of a squat jump at increasing loads (BW, 20 kg, 30 kg, 40 kg). A dowel stick was positioned across the shoulders instead of a barbell for the BW condition. To execute the squat jump (SJ), participants would bend their knees and hips until they lowered themselves to the point of initial height previously measured. They would pause for 2 s in this bottom position before jumping as high as they could with maximal intent. Participants would then land the jump, reset, and repeat for the remaining repetitions.

### 2.3. Training Intervention

Due to the recreational nature of the participants, a 6-week training program was utilized to determine changes in jump performance, as this timeframe has previously yielded significant improvements in jump performance and other reactive tasks [28,29,30,31,32,33]. A combination of resistance and plyometric training was incorporated in the program, as this has also been shown to generate the greatest improvements in jump performance and reactive strength [34,35,36,37]. However, since the maximum strength has been identified as a key determining factor of reactive strength performance [38], a greater emphasis was placed on resistance strength training to target relative strength levels. In doing so, an emphasis on lower-body compound movements, where greater loads could be lifted, was incorporated. For main lifts, between 3 and 5 sets for 5–8 reps were used to emphasize strength development [39], whilst rep schemes for plyometric exercises varied depending on the high or low amplitude nature of the tasks. The training program can be referred to in Table 1.

### 2.4. Statistical Analysis

**Figure 1:**
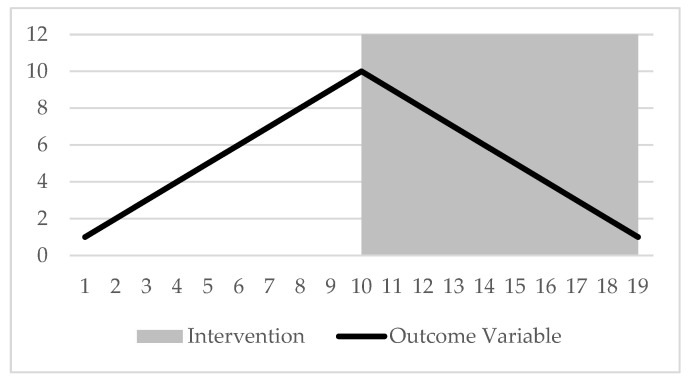
Example of baseline and intervention trends in a SCED with high overlap data and between-phase descriptive statistics but clear trend changes, where intervention appears to reverse the baseline trend.

Traditional SCEDs have used visual inspection to identify changes between baseline and intervention phases [40]. However, with the broader application of SCEDs, non-parametric tests such as the Mann–Whitney U test provided a way to compare baseline and intervention phases. This is because these tests are not bound by the assumptions that traditional parametric tests are [40]. However, this test only works when there is no trend or overlap in baseline data. Trends in baseline data compared to intervention data can result in the same group results; however, on visual inspection, clear and evident differences between phases can be seen. See Figure 1 as an example, where a positive increase in trend is seen during the baseline phase, but a negative decrease in trend is seen in the intervention phase.

However, Parker and colleagues [41] developed a statistical test that controls for data with a trend in the baseline. The Tau-U test combines elements of both Kendall’s rank correlation (Tau) and Mann–Whitney U tests to assess the ‘dominance’ of data by analyzing the nonoverlap between baseline and intervention phase trends whilst correcting for the baseline trend [40]. The Tau-U test has been shown to be a worthwhile statistical option for SCEDs due to its flexibility in effect size, good statistical power, and minimal distributional assumptions [41,42,43,44]. For the purposes of this study, the Tau-U was completed using the following online Tau-U statistical calculator, http://singlecaseresearch.org/calculators/tau-u (accessed 17 April 2024). The process for the analysis of each metric on the calculator was as follows: First, for each participant, an analysis of the baseline trend was completed. Second, if there was no significant baseline trend, baseline and intervention phase data were compared. If there was a significant baseline trend, baseline and intervention phase data were compared with the addition of a baseline control. Third, trends for each participant were reported and then combined to calculate a weighted average, which was reported as the cohort results. Combining the Tau-U results assessed the overall effectiveness of the intervention by minimizing the risk of type 1 and 2 errors. Additionally, the combined results highlighted the influence of individual Tau-U scores on the final outcomes. However, it is also important to highlight the limited generalizability of combining the Tau-U of 4 participants to broader populations. Whilst all studies must consider the representation of the sample size with the broader population, the low sample size nature of the SCED makes this style of research highly specific to the sample size recruited. For an interpretation of the results, the ‘Tau’ score was categorized as follows: 0–0.2 = trivial, 0.2–0.5 = small, 0.5–0.8 = moderate, 0.8–1.0 = large. A positive Tau indicated that the metric increased during the intervention phase, whilst a negative Tau indicated a decrease in the metric output. The *p*-value was set at <0.05.

## 3. Results

**Figure 2:**
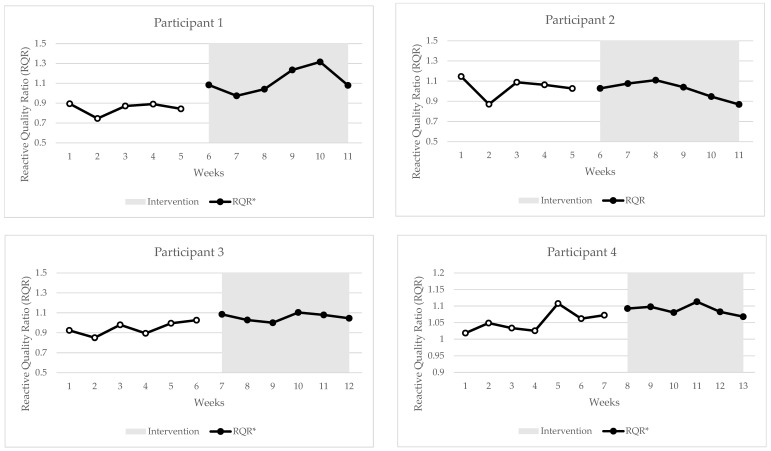
Individual changes in reactive quality ratio; hollow dotted line = baseline phase; RQR = reactive quality ratio; * = statistically significant change in trend *p* < 0.05.

**Figure 3:**
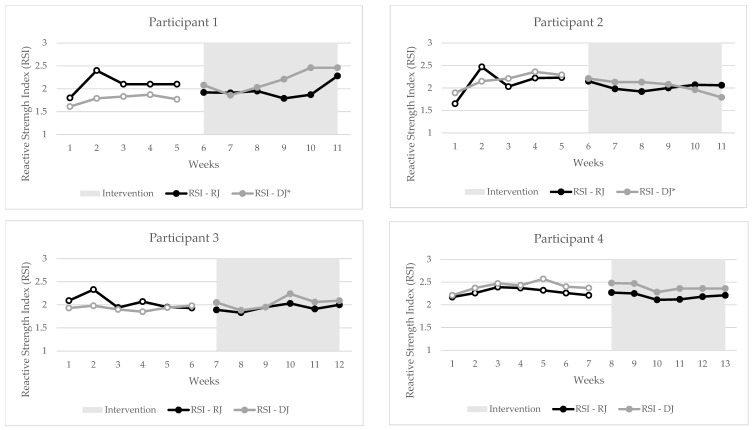
Individual changes in reactive strength index; hollow dotted lines = baseline phase; RSI = reactive strength index; DJ = drop jump; RJ = 10/5 repeated jump; * = statistically significant change in trend, *p* < 0.05.

**Figure 4:**
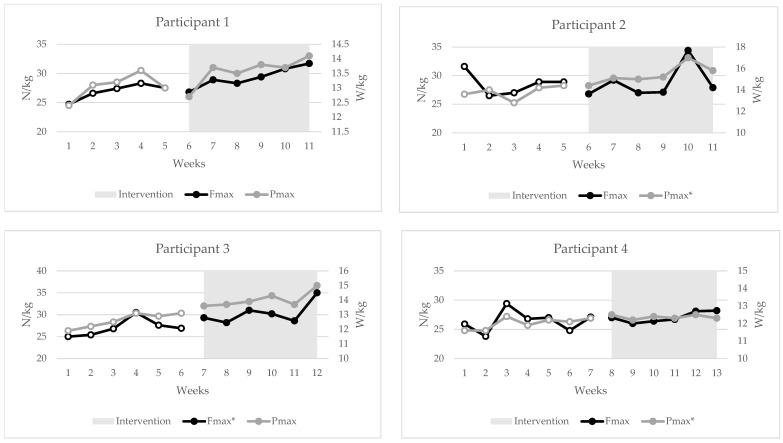
Individual changes in Fmax and Pmax of the Force–Velocity Profile; hollow dotted lines = baseline phase; Fmax = max force; Pmax = max power; * = statistically significant change in trend, *p* < 0.05.

Individual changes in RQR, RSI, and FVP metrics can be seen in Figure 2, Figure 3 and Figure 4. During statistical analysis, the following participants had trends during baseline that were controlled in Tau-U analysis: participant 1—FVP (Fmax, Vmax) and DJ Peak Force; participant 2—DJ (RSI, Mean Force, Peak Power, impulse, GCT and FT), RJ (impulse, Mean Force, Peak Force); participant 3—FVP (jump height at bodyweight, Pmax); participant 4—DJ Landing RFD. As seen in Figure 2, Figure 3 and Figure 4, there were statistically significant changes in trends for the following metrics: participant 1—RQR (Tau = 1.0) and DJ RSI (0.93); participant 2—DJ RSI (−0.83) and Pmax (0.96); participant 3—RQR (0.92) and Fmax (0.78); participant 4—RQR (0.71) and Pmax (0.71). The combined outcomes from this study are found in Table 2.

## 4. Discussion

The main finding of this study was that the SCED design was able to effectively highlight that following a 6-week training block of strength and plyometric training, there was a range of inter-participant differences in the reactive strength trend. At a cohort level, there was no change in the DJ RSI output; however, there was a significant decrease in the RJ RSI (Tau= −0.50), causing a significant, moderate increase in the RQR (Tau = 0.61) following the baseline block. Furthermore, there was a significant small increase in participants’ relative max force (Tau = 0.38) output in their FVP. These findings only partially supported the hypothesis, as it was assumed that changes in the RQR would be a result of an increase in the DJ RSI, not a decrease in the RJ RSI. However, when examining the temporal and kinetic factors of the RJ and DJ, these changes become more complex. The variance in individuals’ responses to the training intervention highlights the intricacies of how individuals adapt to training, which cohort observations alone do not capture. A potential reason for this is the concept of responders and non-responders to exercise.

It is well documented that different individuals will respond differently to physical activity, with genetics, training history, environment, age, and gender all being influencing factors [45,46,47,48]. In regard to resistance training, individuals who have higher type 2 fiber composition, an enhanced insulin-like growth factor (IGF-1) microRNA profile, and higher muscle androgen receptors will experience greater responses to training [49,50,51,52]. Although none of these markers were assessed in the current study, it is not unreasonable to assume that these four participants have different muscle compositions and genetic profiles. Coupled with the wide variances in training history and lifestyle, this would have impacted training response to a 6-week intervention. In this study, participant 2 could be classed as a non-responder due to there being no significant changes in key strength markers of the RQR, RJ RSI, and Fmax and decreased performance in the DJ RSI. As they had close to no prior training history, they could have potentially benefited from a longer intervention and greater volume of work to overcome familiarization with exercises and allow tissue adaptations to occur. Alternatively, participant 1 responded very quickly to the training intervention; however, as seen in the last week, their increasing trend in the RQR stopped in part due to a stagnation in the DJ RSI; therefore, participant 1 may have benefited from the progression of plyometric and strength exercises, emphasizing greater eccentric demand. Additionally, whilst participant 3 exhibited a significant response in the RQR, it was not as exponential as that of participants 1 and 4. This may be attributed to participant 3’s prior training history, which likely resulted in a lower relative training stimulus compared to the others. In contrast, the exponential gains observed in participants 1 and 4 may be explained by the ‘beginner gains’ phenomenon, characterized by increased neuromuscular efficiency, elevated hormone levels, and enhanced muscle adaptation [53]. Furthermore, whilst the cohort RJ RSI had a significant decreasing trend, there was, in fact, no significant decreasing trend at an individual level. This means a practitioner wanting to elicit specific RJ changes in an individual would potentially require a greater volume and focus on low amplitude, extensive plyometrics in their program. Therefore, it is important to consider the individual changes in an intervention, as cohort results alone may not provide an accurate representation of where adaptation has occurred. This highlights the benefits of an SCED as an additional method for sport scientists and training studies to provide more in-depth considerations and applications of training programs. Nonetheless, cohort observations can still provide insights into overall population trends, which were also explored in this study.

At a cohort level, whilst the overall RSI output decreased in the RJ, the underlying temporal and kinetic metrics showed that this decrease in performance may be because of inefficiency in controlling newly developed force expression abilities, rather than an overall decrease in performance. Compared to the baseline phase, participants had a small, significant increase in the FT (Tau = 0.43), attributed to a small increase in impulse (Tau = 0.46). This may indicate that individuals were exerting more force into the ground upon contact to jump higher in the air. However, in spending more time in the air, they were losing reactivity by spending more time on the ground between hops to counter the increased eccentric demand. This was seen with a moderate increase in the GCT (Tau= 0.76) and decrease in all other examined kinetic variables of the RJ (Peak Force −0.53, Average Force −0.46, Landing RFD −0.58, Active Stiffness −0.67, and Peak Power −0.42). This increased eccentric demand would cause a longer amortization time, which is the time delay period from overcoming eccentric work to concentric work during ground contact. A longer amortization time reduces the engagement of the stretch reflex and reduces the ability to utilize stored energy, which is dissipated as heat, thus leading to reduced reactive performance [54]. One could argue what came first, increasing jump height or decreasing reactivity; however, the technique of the RJ has participants starting with a jump, where the landing equates to the first ground contact. Therefore, the GCT is always impacted by the eccentric demands of the preceding jump/hop. Another potential reason for this loss in RJ reactivity following training is that tendons take 8–12 weeks for changes in cross-sectional area (CSA), stiffness, and Young’s modulus [55,56,57]. In contrast, changes in muscle CSA and neuromuscular drive have been observed in as little as 2–4 weeks [58,59,60]. With only a 6-week intervention phase, adaptations in muscle strength may have occurred (which is seen with increases in the FT of the RJ and FVP metrics); however, the Achilles tendon may not have had enough time to adapt. Furthermore, exposure to the low amplitude skipping training once a week may not have been an ample stimulus to improve the neuromuscular coordination of the RJ. Low amplitude reactive strength tasks, such as skipping, often challenge jump rhythm and timing, compared to high amplitude reactive strength tasks, which challenge strength demands due to the higher kinetic output [16,17,61]. Therefore, the fact that there was no decrease in the DJ may have been controlled by the greater focus on strength development in the training program. This emphasis on strength development may have also increased muscle pre-activation and stretch reflex sensitivity of type 2 muscles, such as the gastrocnemius and quads, which have greater involvement in the DJ than RJ due to the higher stretch velocities in the DJ [62,63].

The different kinetic responses the DJ and RJ had to the intervention also provides an indication that the two tests represent different underlying reactive qualities, with the intervention appearing to elicit greater responses to the low amplitude nature of the RJ. This was not seen in the DJ. Whilst it was hypothesized that strength training would impact reactive performance, it was thought by the authors that this would be more targeted towards the DJ due to its high eccentric demand and force requirements of the test. A potential explanation that the RJ was more sensitive to change may reflect the strength levels of the individuals. The recreational participants in this study had relative strength levels of Fmax = 27–28 N/kg, which compared to professional rugby league players (Fmax = 55–65 N/kg) [64], was low. This may mean that their strength levels are within an optimal range that translates to RJ performance, but not strong enough to translate to the high strength requirements of the DJ. However, these thresholds have not been determined.

Whilst strength levels for these participants were low, it was evident that this intervention did have significant positive results in increasing relative strength and power levels from baseline. The FVP showed small positive improvements in Fmax (27–28.3 N/kg) values, with moderate improvements in Pmax (12.8–13.7 W/kg). In the case of the FVP, the participants became more efficient at generating force in the ballistic movement of the SJ. This can be seen by the moderate increases in the JH at bodyweight (27.8–30.5 cm), and with an additional 40 kg load (14.6–16.6 cm). These results were expected, as the primary strength exercises of the intervention program were squats and deadlifts, which primarily depend on the hip and knee joints [65,66,67]. Naturally, this would have a high transfer rate to the SJ used to determine the FVP due to the similar motor patterns and muscles recruited, primarily the glutes, quads, and hamstrings [66]. Furthermore, Pmax had the biggest trend change out of all FVP metrics (Tau–Pmax = 0.75 vs. Fmax = 0.38 vs. Vmax = 0.02), which, whilst a reflection of adaptation to training, may also be due to the specificity of the SJ as a power exercise on the force–velocity curve [68]. No improvements in Vmax were found; however, no velocity-specific training was undertaken.

The SCED is an effective way to measure changes in the RQR observed from a training intervention in a population with a small sample size. Furthermore, this study design also highlights the impact of the sample size, inter-participant variation, and variability in participant outcome scores on the ability to determine statistically significant effects during SCED studies. For example, participants 1 and 3 had a large increase in the RQR trend through the intervention (Tau = 1.0 and 0.93, respectively), while participant 4 had a moderate (Tau = 0.71) increase. However, participant 2 had a non-significant negative change in the trend, which, overall, decreased the training effect on the combined RQR results (Tau = 0.61). Further examples are seen with underlying changes in the RSI, with all participants showing non-significant moderate negative changes in the RJ. However, due to low inter-participant variation in results, this improved the power of the results in the combined analysis to show a significant moderate decrease (Tau = −0.50). The RSI for the DJ showed the most variance between individuals, with participant 1 showing a large positive change (Tau = 0.9), participant 2 showing a large negative change (Tau = −0.83), and participants 3 and 4 showing no significant changes (Tau = 0.61 and −0.26, respectively). Interestingly, the two strongest participants, participants 1 and 3, who also displayed greater than moderate increases in Fmax trends (Tau = 0.5 and 0.78, respectively), were the only two that had a positive increase in the DJ RSI, be it a significant change or not. This once again suggests the importance of increases in strength levels to be able to effectively express reactive strength during an intensive plyometric exercise such as the DJ. Nonetheless, it is important for practitioners to understand the difference between individual and cohort responses to a training program, as cohort results may not always capture the full representation of an individual’s changes, as seen in this study.

There are certain considerations or areas of future research when analyzing the results from this study. First, the participants recruited in this study were recreationally active or relatively untrained males. Future research looking at the changes in reactive strength in more advanced and specific athlete populations could prove beneficial in determining whether greater inter-participant variance occurs in these populations. Additionally, the observation of females could provide further insights into the impact of resistance training on reactive strength and sensitivity to adaptation. For instance, the lower Achilles tendon stiffness in females than males could potentially impact the RJ and DJ RSI response [69]. This would further support the importance of monitoring individual responses to training. Unfortunately, no females expressed interest in participating during recruitment for the current study. Second, future research could conduct a longer intervention phase of 10–12 weeks to determine if this would elicit a greater change in RJ RSI. This would highlight that more time is needed for tendon adaptations to occur and confirm a greater involvement of elastic properties in the RJ over the DJ. Third, obtaining optimal DJ height to individualize intervention prescription for the DJ exercise may have created a greater training stimulus for the participants, potentially having greater transfer to the DJ test. Fourth, whilst a combination of both strength and plyometric training was used in this study, a greater emphasis was placed on strength training due to previous research highlighting the positive influence of relative strength on DJ RSI [38]. However, future research could examine the effects of specific low amplitude vs. high amplitude plyometric training exposure and their impacts on changing the RQR. Fifth, as previously mentioned, it is important to consider the generalizability of applying SCEDs to broader populations due to the small sample size. Whilst the SCED is highly specific in identifying individual changes following intervention, practitioners would benefit from using benchmark data determined from cohort studies before utilizing a SCED to monitor an individual’s response to training.

## 5. Conclusions

The findings from this study highlight that non-concurrent, multiple baseline SCEDs provide unique insights by highlighting the variance of change between individuals following a 6-week training intervention. Whilst some individuals displayed significant positive changes in reactive strength and FVP performance, others were not so evident. This may be a result of high and low responders to exercise, and practitioners should adjust training programs to reflect these individual differences. Additionally, this study highlighted that low amplitude and high amplitude reactive strength will respond differently to training interventions, and the RQR is sensitive enough to reflect these differences in recreationally active individuals. However, the relationships underpinning these changes are complex.

## Figures and Tables

**Table 1:** Training intervention.

**Day 1.**	**Week 1**	**Week 2**	**Week 3**	**Week 4**	**Week 5**	**Week 6**
	**Reps**	**Reps**	**Reps**	**Reps**	**Reps**	**Reps**
Warm-Up						
Stationary Bike	5 min	5 min	5 min	5 min	5 min	5 min
BW Split Squat	1 × 5 ea	1 × 5 ea	1 × 5 ea	1 × 5 ea	1 × 5 ea	1 × 5 ea
Glute Bridges	1 × 10	1 × 10	1 × 10	1 × 10	1 × 10	1 × 10
BW CMJ	1 × 3	1 × 3	1 × 3	1 × 3	1 × 3	1 × 3
Testing Protocol						
10/5 Repeated Jump	1 × 10	1 × 10	1 × 10	1 × 10	1 × 10	1 × 10
Drop Jump @ 45 cm	1 × 3	1 × 3	1 × 3	1 × 3	1 × 3	1 × 3
Loaded Squat Jump	4 × 2	4 × 2	4 × 2	4 × 2	4 × 2	4 × 2
Main Session						
Barbell Box Squat	5 × 5	5 × 5	5 × 5	5 × 5	5 × 5	5 × 5
Skipping	3 × 20	3 × 30	3 × 30	3 × 40	3 × 40	3 × 40
SL Calf Raise—Straight Knee	2 × 10	2 × 10	2 × 15	2 × 15	2 × 20	2 × 20
**Day 2.**	**Week 1**	**Week 2**	**Week 3**	**Week 4**	**Week 5**	**Week 6**
	**Reps**	**Reps**	**Reps**	**Reps**	**Reps**	**Reps**
Warm-Up						
Stationary Bike	5 min	5 min	5 min	5 min	5 min	5 min
BW Split Squat	1 × 5 ea	1 × 5 ea	1 × 5 ea	1 × 5 ea	1 × 5 ea	1 × 5 ea
Glute Bridges	1 × 10	1 × 10	1 × 10	1 × 10	1 × 10	1 × 10
BW CMJ	1 × 3	1 × 3	1 × 3	1 × 3	1 × 3	1 × 3
Main Session						
Box Squat/Deadlift	4 × 6	4 × 6	4 × 6	4 × 6	4 × 6	4 × 6
Drop Jump	3 × 3 @ 30 cm	3 × 3 @ 30 cm	3 × 3 @ 30 cm	3 × 3 @ 40 cm	3 × 3 @ 40 cm	3 × 3 @ 40 cm
Barbell Romanian Deadlift	3 × 8	3 × 8	3 × 8	3 × 8	3 × 8	3 × 8
Split Squat	3 × 8 ea	3 × 8 ea	3 × 8 ea	3 × 8 ea	3 × 8 ea	3 × 8 ea
SL Calf Raise—Bent Knee	2 × 10	2 × 10	2 × 15	2 × 15	2 × 20	2 × 20

BW—bodyweight; CMJ—countermovement jump; SL—single leg; ea—each side.

**Table 2:** SCED combined descriptive results.

Metric	Baseline		Intervention		Tau	*p*-Value	CI 95%
	Median	IQR	Median	IQR			
Bodyweight (kg)	76	71–91	77	70–88	0.60	**<0.001**	0.252–0.950
Reactive Quality Ratio	1.02	0.89–1.06	1.08	1.03–1.10	0.61	**<0.001**	0.268–0.959
10/5 RSI	2.15	2.01–2.32	2.00	1.92–2.14	−0.50	**0.005**	−0.845–−0.154
DJ RSI	1.98	1.87–2.37	2.13	2.03–2.34	0.03	0.859	−0.317–0.380
10/5 Repeated Jump							
GCT (ms)	191	174–205	206	185–224	0.76	**<0.001**	0.415–1.000
FT (ms)	415	359–452	431	376–474	0.43	**0.017**	0.076–0.775
Impulse (Ns)	448	413–480	479	402–503	0.46	**0.009**	0.110–0.808
Peak Force (N)	4414	3791–4923	3914	3694–4490	−0.53	**0.003**	−0.881–−0.183
Average Force (N)	2394	2133–2577	2191	2037–2450	−0.46	**0.009**	−0.808–−0.110
Landing RFD (N/s)	51,315	38,627–69,166	44,754	35,225–53,221	−0.58	**0.001**	−0.928–−0.230
Active Stiffness (N/m)	29,360	26,321–217,710	25,622	21,710–34,047	−0.67	**<0.001**	−1.000–−0.323
Peak Power (W)	5060	4622–5887	4621	4356–5360	−0.42	**0.019**	−0.767–−0.069
Drop Jump							
GCT (ms)	213	206–219	218	207–222	0.41	**0.021**	0.060–0.758
FT (ms)	430	409–482	462	452–495	0.59	**<0.001**	0.240–0.938
Impulse (Ns)	621	543–671	613	563–644	0.25	0.164	−0.101–0.597
Peak Force (N)	4422	3354–5613	3825	3448–4934	−0.13	0.45	−0.483–0.214
Average Force (N)	2187	1835–2273	2138	1898–2261	0.06	0.724	−0.286–0.411
Landing RFD (N/s)	4422	3354–5613	3825	3448–4934	0.07	0.699	−0.280–0.418
Active Stiffness (N/m)	19,506	16,305–22,797	18,804	17,122–21,297	−0.06	0.730	−0.410–0.287
Peak Power (W)	13,410	11,036–14,094	13,284	11,628–13,554	−0.25	0.167	−0.595–0.102
Passive Stiffness (N/m)	12,561	10,990–21,770	13,713	9665–16,224	−0.13	0.479	−0.475–0.222
Force–Velocity Profile							
JH @ Bodyweight (cm)	27.8	26.6–30.4	30.5	28.2–35.4	0.63	**<0.001**	0.288–0.980
JH @ + 40 kg (cm)	14.6	12.5–16.2	16.6	14.3–18.5	0.68	**<0.001**	0.331–1.000
Fmax (N/kg)	27	25.9–28.3	28.3	27–30	0.38	**0.03**	0.036–0.728
Vmax (m/s)	1.9	1.8–2.0	1.9	1.8–2.0	0.02	0.905	−0.324–0.366
Pmax (W/kg)	12.8	12.2–13.2	13.7	12.6–14.9	0.75	**<0.001**	0.405–1.000

SCED = single-case experimental design; IQR = interquartile range; CI = confidence interval; RSI = reactive strength index; GCT = ground contact time; FT = flight time; RFD = rate of force development; JH = jump height; Fmax = theoretical max force; Vmax = theoretical max velocity; Pmax = theoretical peak power; **Bold** = *p* < 0.05.

## Data Availability

Data are unavailable for public access due to ethical restrictions highlighted by the ethics committee.

## Abbreviations

The following abbreviations are used in this manuscript:

RSI	Reactive Strength Index
RQR	Reactive Quality Ratio
GCT	Ground Contact Time
FT	Flight Time
FVP	Force Velocity Profile
DJ	Drop Jump
RJ	10/5 Repeated Jump
